# Uncovering the molecular mechanisms between heart failure and end-stage renal disease *via* a bioinformatics study

**DOI:** 10.3389/fgene.2022.1037520

**Published:** 2023-01-10

**Authors:** Rutao Bian, Xuegong Xu, Weiyu Li

**Affiliations:** Zhengzhou Traditional Chinese Medicine Hospital, Zhengzhou, China

**Keywords:** heart failure, end-stage renal disease, hub gene, bioinformatics analysis, immune infiltration

## Abstract

**Background:** Heart failure (HF) is not only a common complication in patients with end-stage renal disease (ESRD) but also a major cause of death. Although clinical studies have shown that there is a close relationship between them, the mechanism of its occurrence is unclear. The aim of this study is to explore the molecular mechanisms between HF and ESRD through comprehensive bioinformatics analysis, providing a new perspective on the crosstalk between these two diseases.

**Methods:** The HF and ESRD datasets were downloaded from the Gene Expression Omnibus (GEO) database; we identified and analyzed common differentially expressed genes (DEGs). First, Gene Ontology (GO), Kyoto Encyclopedia of Genes and Genomes (KEGG), and gene set variation analyses (GSVA) were applied to explore the potential biological functions and construct protein−protein interaction (PPI) networks. Also, four algorithms, namely, random forest (RF), Boruta algorithm, logical regression of the selection operator (LASSO), and support vector machine-recursive feature elimination (SVM-RFE), were used to identify the candidate genes. Subsequently, the diagnostic efficacy of hub genes for HF and ESRD was evaluated using eXtreme Gradient Boosting (XGBoost) algorithm. CIBERSORT was used to analyze the infiltration of immune cells. Thereafter, we predicted target microRNAs (miRNAs) using databases (miRTarBase, TarBase, and ENOCRI), and transcription factors (TFs) were identified using the ChEA3 database. Cytoscape software was applied to construct mRNA−miRNA−TF regulatory networks. Finally, the Drug Signatures Database (DSigDB) was used to identify potential drug candidates.

**Results:** A total of 68 common DEGs were identified. The enrichment analysis results suggest that immune response and inflammatory factors may be common features of the pathophysiology of HF and ESRD. A total of four hub genes (BCL6, CCL5, CNN1, and PCNT) were validated using RF, LASSO, Boruta, and SVM-RFE algorithms. Their AUC values were all greater than 0.8. Immune infiltration analysis showed that immune cells such as macrophages, neutrophils, and NK cells were altered in HF myocardial tissue, while neutrophils were significantly correlated with all four hub genes. Finally, 11 target miRNAs and 10 TFs were obtained, and miRNA−mRNA−TF regulatory network construction was performed. In addition, 10 gene-targeted drugs were discovered.

**Conclusion:** Our study revealed important crosstalk between HF and ESRD. These common pathways and pivotal genes may provide new ideas for further clinical treatment and experimental studies.

## Introduction

Heart failure (HF) is a common clinical cardiovascular disease that affects more than 26 million people worldwide (Savarese and Lund, 2017). One of the most common causes of heart failure is ischemic heart disease, which causes a loss of myocardial tissue and contractility (Severino et al., 2020). At this stage, the main goal of the pharmacological treatment of this disease is to relieve symptoms and improve residual cardiac functions. The prevalence of HF increases significantly as the renal function deteriorates (Silverberg et al., 2004), reaching 65%–70% in end-stage renal disease (ESRD). Conversely, the risk of HF is 15.8 times higher in patients with progressive chronic kidney disease (Schefold et al., 2016). On the other hand, abnormal renal function may affect the application of HF therapeutic agents, increase the risk of nephrotoxicity in HF treatment, and impair the patient’s response to diuretics. These results suggest that several susceptibility factors in kidney diseases may trigger the development of HF.

Studies have shown that the myocardium in patients with ESRD exhibits characteristic changes, often accompanied by pathological myocardial fibrosis and cardiac hypertrophy (Alhaj et al., 2013). Recent scientific work has identified several possible pathophysiological mechanisms, including hemodynamic disturbances, excessive activation of the RAAS, water and sodium retention, chronic inflammatory states, metabolic acidosis, reduced cytokine clearance, insulin resistance, oxidative stress, and post-translational modification of blood-borne molecules such as lipoproteins (Zoccali et al., 2017; Rangaswami and McCullough, 2018; Costanzo, 2020). At this stage, mechanistic studies have mostly focused on the excessive activation of the RAAS and thus cardiac remodeling (Dounousi et al., 2021). Although some studies have found that abnormalities in immune cells in ESRD patients promote cardiovascular disease, the direct effects of abnormal ratios of immune cells in the blood and alterations in inflammatory factors on the myocardial tissue in ESRD patients are often overlooked. Despite there being strong clinical and epidemiological evidence of the crosstalk between HF and ESRD, the exact mechanisms explaining the coexistence of the two diseases remain unclear. A better understanding of the relationship between them is therefore essential for the development of detection and management.

With the advancement of science and technology, bioinformatics approaches have enabled us to gain a deeper understanding of the biological processes of disease pathology at the genetic level. However, the common diagnosis and interlinked genes in HF and ESRD are unclear. Therefore, this study used bioinformatics methods to screen the biomarkers, which may serve to provide new insights into the biological mechanisms of these two diseases.

## Materials and methods

### Data source

We searched the Gene Expression Omnibus (GEO) database (http://www.ncbi.nlm.nih.gov/geo/) for gene expression datasets using the terms “heart failure” or “end stage renal disease.” The following criteria were then used to further screen the dataset: 1) studies that included cases *versus* healthy controls; 2) heart failure samples were obtained from ischemic HF heart tissue; 3) ESRD samples were obtained from blood samples; and 4) the sample size was greater than 30. Finally, GSE57338, GSE5604, and GSE48116 were identified as HF datasets, and GSE37171, GSE9709, and GSE67401 were identified as ESRD datasets. The information on each dataset is summarized in Table 1.

**TABLE 1:** Summary of the four GEO datasets involving HF and ESRD patients.

Index	GSE number	Platform	Sample	Source type	Disease	Group
1	GSE57338	GPL11532	95 ischemic HF patients and 136 normal subjects	Expression profiling by array	HF	Discovery cohort
2	GSE5406	GPL96	108 ischemic HF patients and 16 normal subjects	Expression profiling by array	HF	Validation cohort
3	GSE48116	GPL9115	15 ischemic HF patients and 15 normal subjects	Expression profiling by high-throughput sequencing	HF	Validation cohort
4	GSE37171	GPL570	75 patients and 40 normal subjects	Expression profiling by array	ESRD	Discovery cohort
5	GSE97709	GPL17303	28 patients and 12 normal subjects	Expression profiling by high-throughput sequencing	ESRD	Validation cohort
6	GSE67401	GPL9115	58 patients and 22 normal subjects	Expression profiling by high-throughput sequencing	ESRD	Validation cohort

HF, heart failure; ESRD, end-stage renal disease.

### Identification of DEGs

In GSE57338 and GSE37171 datasets, gene expression was analyzed for subsequent analyses, and the “limma” package (Ritchie et al., 2015) was used to calculate the differentially expressed genes (DEGs), with *p*-value <.05 and |log_2_ fold-change (LogFC)> 0.5 considered statistically significant. Subsequently, the batch effects were removed using the “ComBat” package for the next stage of the analysis. The “ggplot2” (Ito and Murphy, 2013) and “pheatmap” packages were used to plot DEGs for visualizing different datasets.

### Enrichment analyses

To reveal the potential functions of common DEGs in HF and ESRD, Gene Ontology (GO) (Carbon et al., 2009) and Kyoto Encyclopedia of Genes and Genomes (KEGG) (Kanehisa and Goto, 2000) analyses of common DEGs were performed to explore the functions of DEGs. In the present study, a protein−protein interaction (PPI) network of DEGs was constructed using the STRING database (Szklarczyk et al., 2019).

### Gene set variation analysis enrichment analysis

We performed GSVA (Hänzelmann et al., 2013) enrichment analysis using the “GSVA” R package to explore the potential biological functions involved in HF and ESRD. The gene list of pathways was collected by integrating the Molecular Signatures Database (MSigDB, v7.0) (Subramanian et al., 2005). *p*-values <.05 indicate the statistically significant differences.

### Hub gene screening and validation by multiple machine learning methods

We jointly used four algorithms, namely, random forest (RF) (Alakwaa et al., 2018), Boruta algorithm (Kursa, 2014), logical regression of the selection operator (LASSO) (Alhamzawi and Ali, 2018), and support vector machine-recursive feature elimination (SVM-RFE) (Lin et al., 2012), to screen potential marker genes in HF and ESRD datasets. RF algorithm is a supervised machine learning algorithm that classifies features based on their processing and variables. The Boruta package uses the Boruta algorithm for significant feature gene extraction. The glmnet package is used for LASSO regression, where the best λ is determined by the least binomial deviation with tenfold cross-validation. SVM-RFE is a supervised machine learning algorithm that ranks different features based on differences in predictive power (Sanz et al., 2018). The crossover genes obtained by four machine learning methods have high accuracy in determining diagnostic gene signals (Gao et al., 2021). Meanwhile, HF (GSE5406) and ESRD (GSE97709) validation datasets were used to draw the expression of hub genes.

### Evaluation of the hub gene diagnostic value

eXtreme Gradient Boosting (XGBoost) (Ogunleye and Wang, 2020) is a commonly used supervised integrative learning algorithm that can use the expression values of pivotal genes as the feature values for XGBoost model training. First, we select the HF dataset (GSE57338) and ESRD dataset (GSE37171) as the training sets, and the evaluation is performed on the datasets of HF (GSE48116) and ESRD (GSE67401). The prognostic efficiency was evaluated by the receiver operating characteristic (ROC) curve, precision–recall (PR) curve, and area under the curve (AUC).

### Analysis of immune cell infiltration

We used the CIBERSORT package (Newman et al., 2015) to quantify the level of immune cell infiltration in each sample and to assess the effect of hub genes on immune infiltration in the HF myocardial tissue. The Wilcoxon test was used to analyze the immune scores, the “vioplotR” package was employed to visualize the results, and *p* <.05 was considered significant.

Construction of the miRNA−mRNA−TF regulatory network

The miRTarBase database (Chou et al., 2018), TarBase database (Karagkouni et al., 2018), and ENOCRI database (Li et al., 2014) were used to discover potential miRNAs. Furthermore, we overlapped predicted target miRNAs and constructed a miRNA–target gene regulatory network. The ChIP-X Enrichment Analysis 3 (ChEA3) (Keenan et al., 2019) verified the targets of TFs, and the top 10 TFs were selected as target TFs. Cytoscape (Shannon et al., 2003) was used to visualize the miRNA−mRNA−TF regulatory network.

### Evaluation of candidate drugs

The Enrichers platform was used to identify the relationship between drug molecules and hub genes, and the data were obtained from the Drug Signatures Database (DSigDB, http://tanlab.ucdenver.edu/DSigDB). We retrieved potential drugs for the hub genes.

## Results

### Identification of DEGs

**FIGURE 1:**
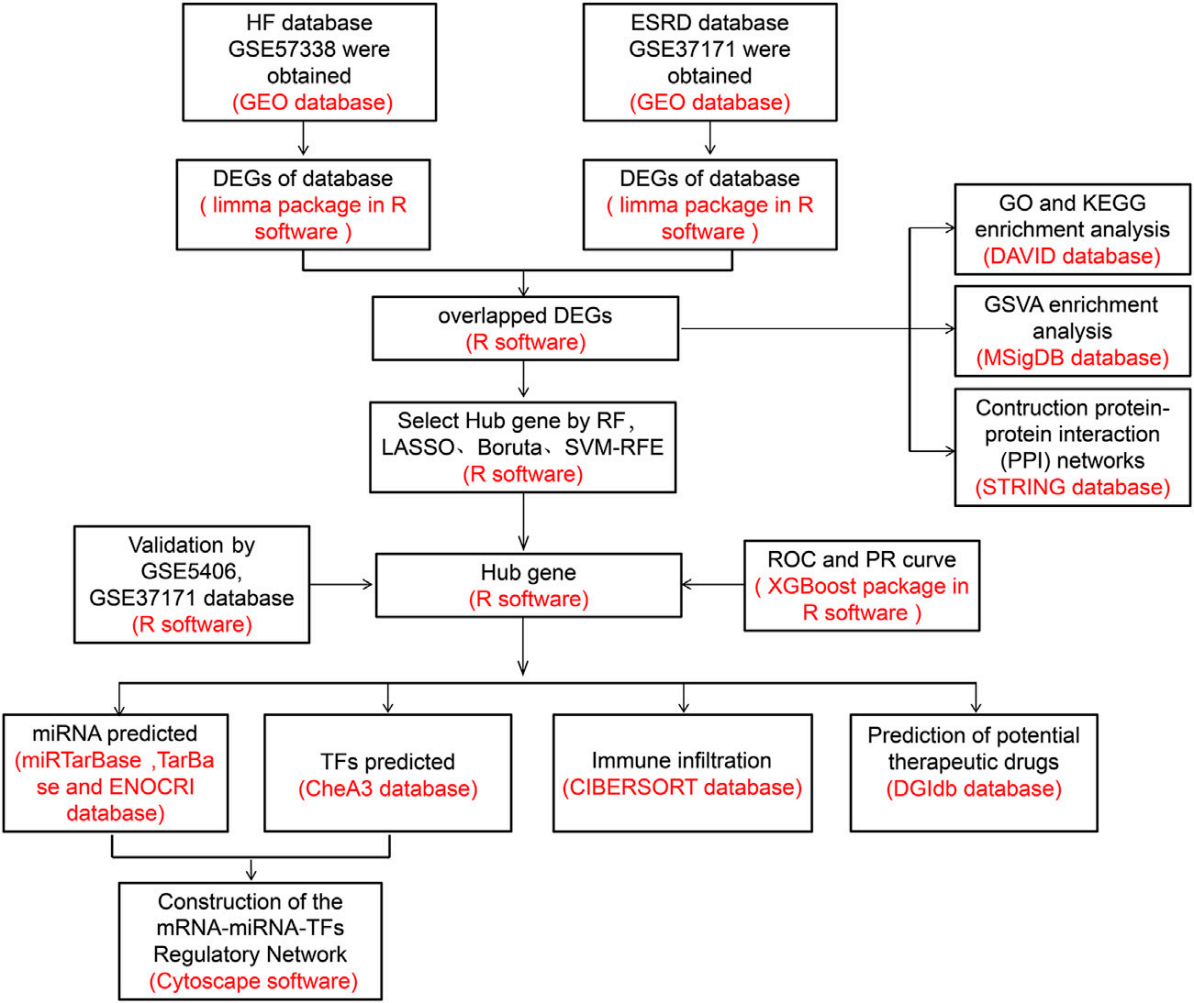
Study design of this research.

**FIGURE 2:**
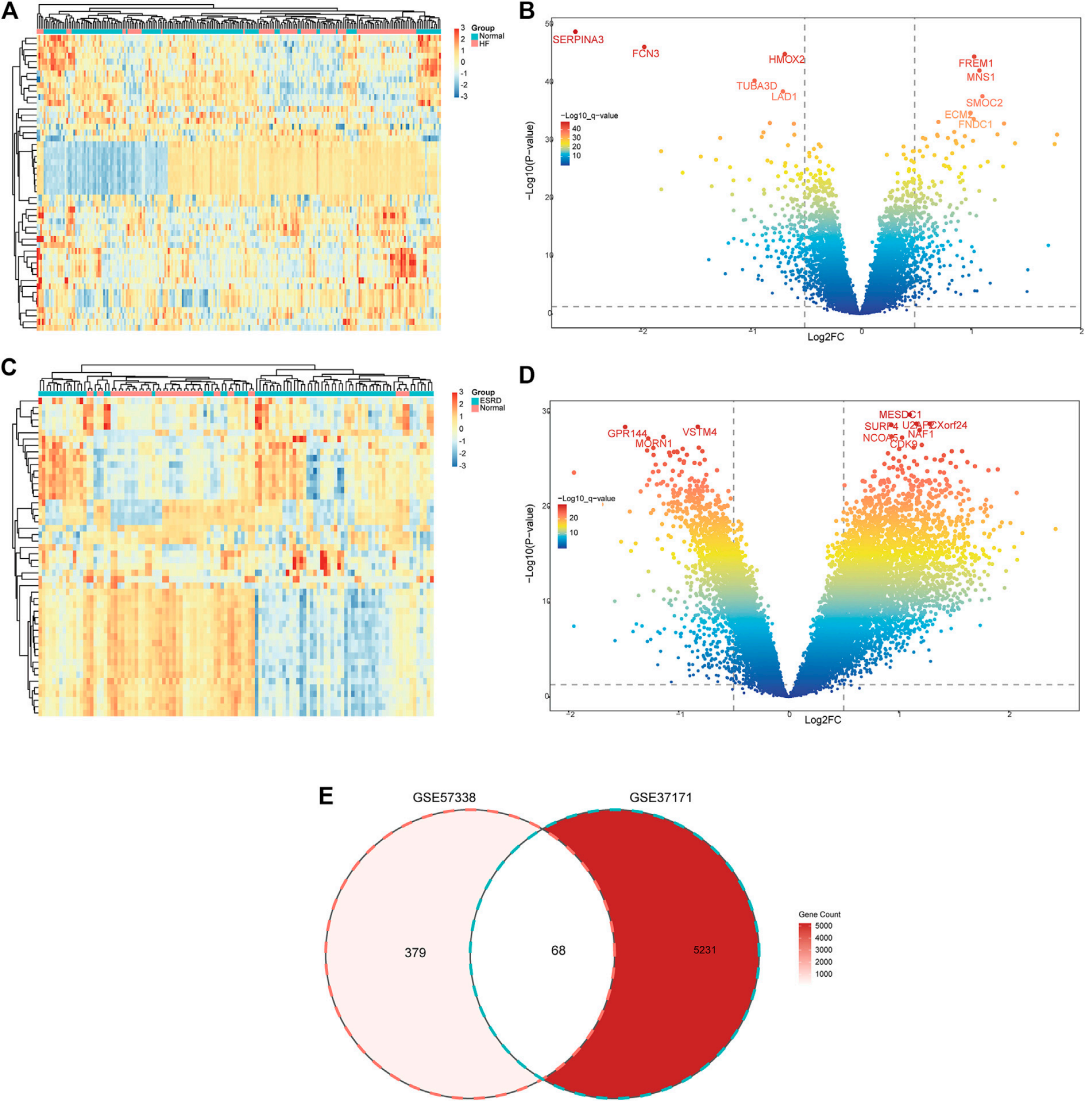
Identification of common DEGs. **(A)** Heatmap of the top 50 DEGs in GSE57338. **(B)** Volcanic plots of GSE57338. **(C)** Heatmap of the top 50 DEGs in GSE37171. **(D)** Volcanic plots of GSE37171. **(E)** Venn diagram of common DEGs in HF and ESRD. HF, heart failure; ESRD, end-stage renal disease.

The flow chart of the study design is shown in Figure 1. A total of 447 DEGs were found in the screened HF and healthy subjects in the dataset GSE57338, including 250 upregulated and 197 downregulated genes (Figures 2A, B). There were 5,299 DEGs in ESRD patients compared to healthy controls, including 4,013 upregulated and 1,286 downregulated genes (Figures 2C, D). A total of 68 common DEGs were identified after taking the intersection of the Venn diagrams (Figure 2E).

### Functional enrichment analyses and the PPI network

**FIGURE 3:**
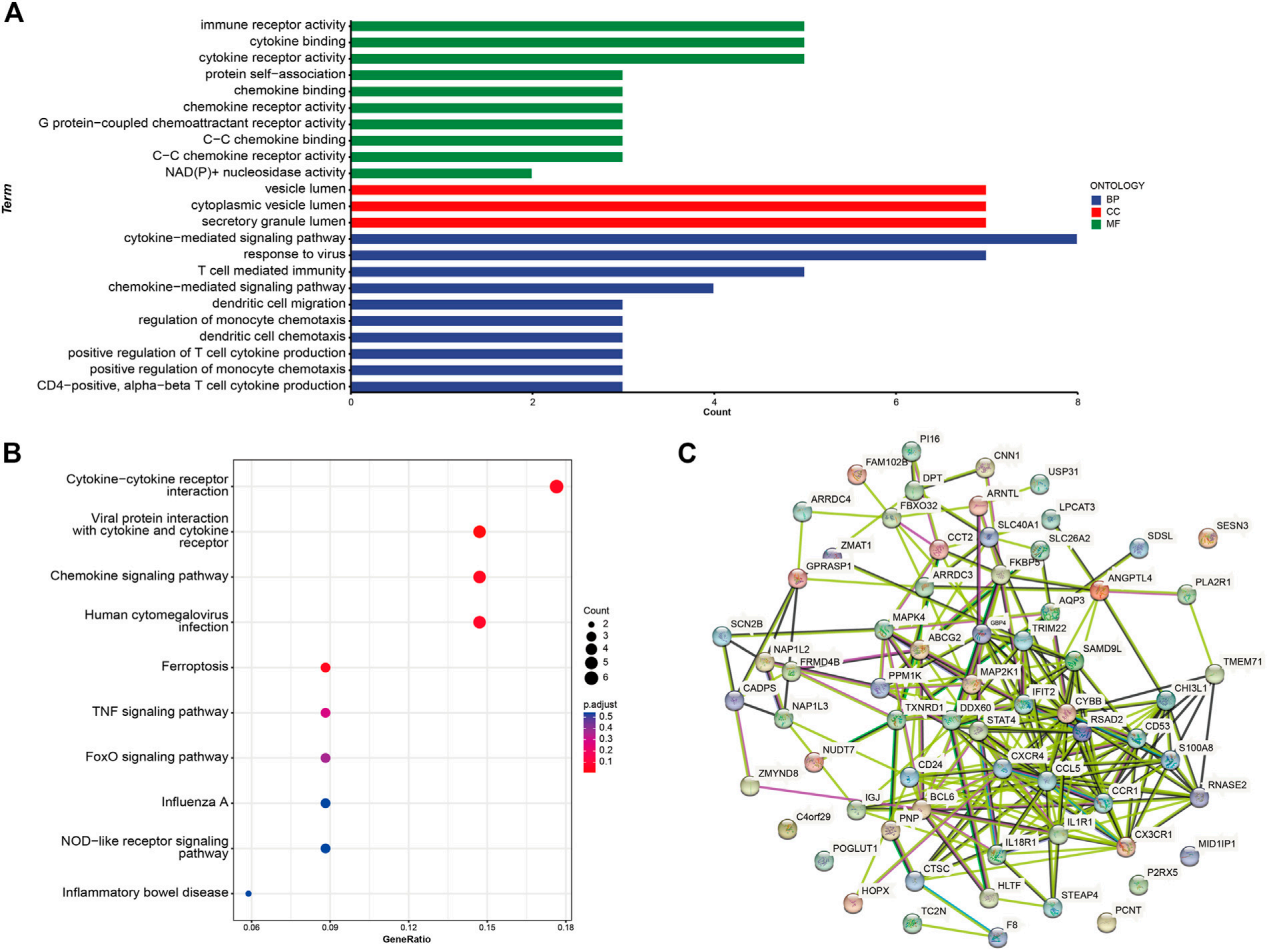
Enrichment analyses for common DEGs. **(A)** GO enrichment analysis. **(B)** KEGG enrichment analysis. **(C)** PPI network; the nodes represent proteins, and the edges represent interactions. GO, Gene Ontology; BP, biological processes; CC, cellular components; MF, molecular function; KEGG, Kyoto Encyclopedia of Genes and Genomes; PPI, protein−protein interaction.

The potential functions and pathways of these common DEGs were detected through the GO and KEGG clustering analyses. The GO term enrichment analysis includes biological processes (BPs), cellular components (CCs), and molecular functions (MFs). In BP, module genes were significantly enriched in immune receptor activity, cytokine binding, cytokine receptor activity, protein self-association, chemokine binding, chemokine receptor activity, and G protein-coupled chemoattractant receptor activity. In MF, module genes were significantly enriched in the cytokine-mediated signaling pathway, response to the virus, T-cell-mediated immunity, chemokine-mediated signaling pathway, dendritic cell migration, and regulation of monocyte chemotaxis. In CC, module genes were significantly enriched in the vesicle lumen, cytoplasmic vesicle lumen, and secretory granule lumen (Figure 3A). KEGG analysis showed enrichment in the cytokine−cytokine receptor interaction, viral protein interaction with cytokine and cytokine receptor, chemokine signaling pathway, human cytomegalovirus infection, ferroptosis, TNF signaling pathway, and FOXO signaling pathway (Figure 3B). KEGG analysis also showed that processes such as immune factors and chemokine and immune cell regulation were mainly enriched. We used the STRING online server to construct a PPI network (Figure 3C)//.

### GSVA enrichment analysis

**FIGURE 4:**
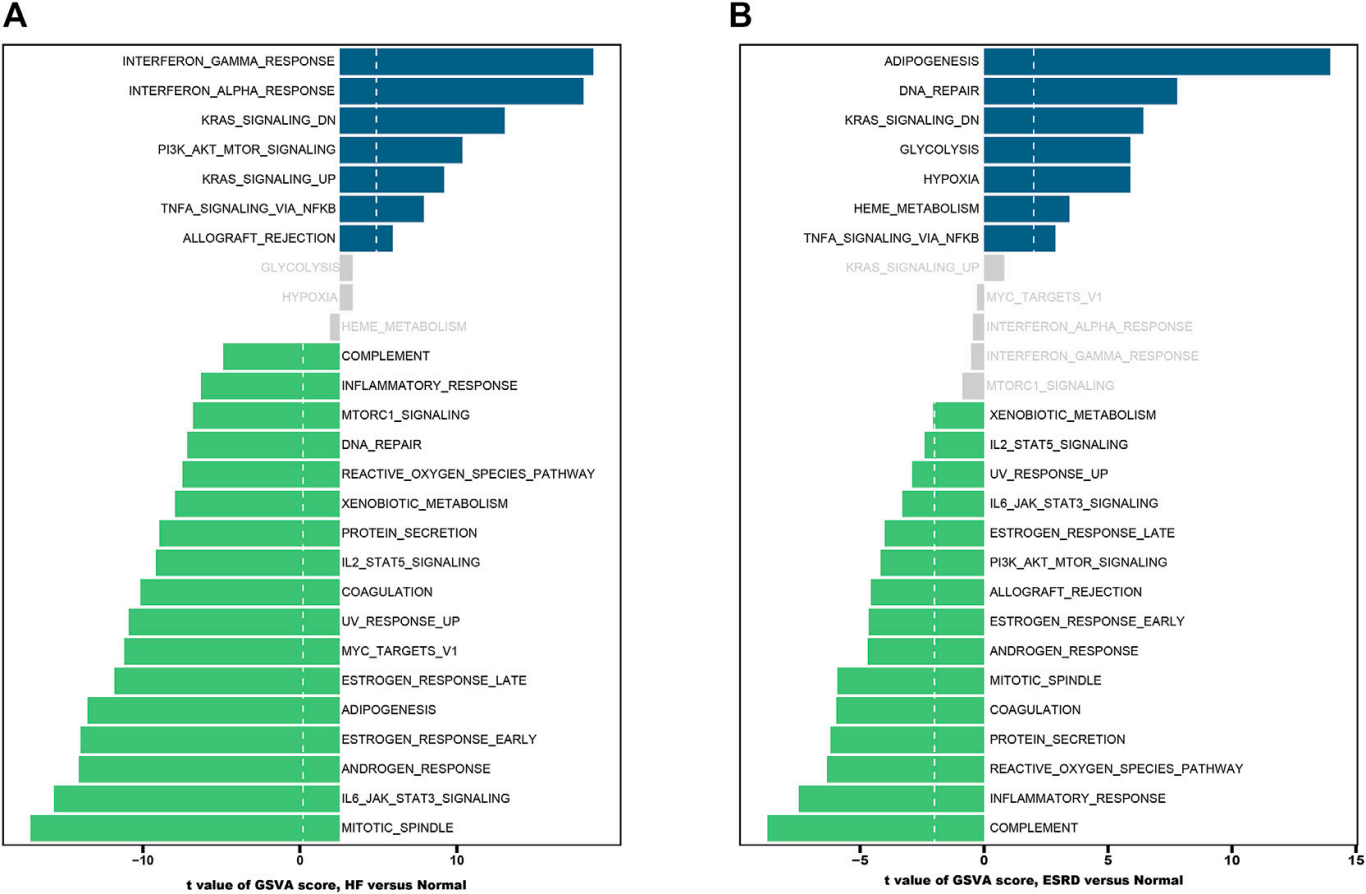
Functional and pathway analysis by GSVA enrichment analysis in HF and ESRD. **(A)** Significant related biological pathways in HF were obtained by GSVA. **(B)** Significant related biological pathways in ESRD were obtained by GSVA. The blue bars represent high expression, and the green bars represent low expression. HF, heart failure; ESRD, end-stage renal disease; GSVA, gene set variation analysis.

The results of GSVA enrichment analysis revealed that immune‐related response, interferon gamma response, interferon alpha response, and KRAS signaling DN were positively correlated with HF (Figure 4A). Meanwhile, the high expression of adipogenesis, DNA repair, and KRAS signaling DN were found to be related with ESRD (Figure 4B).

### Identification and validation of hub genes

**FIGURE 5:**
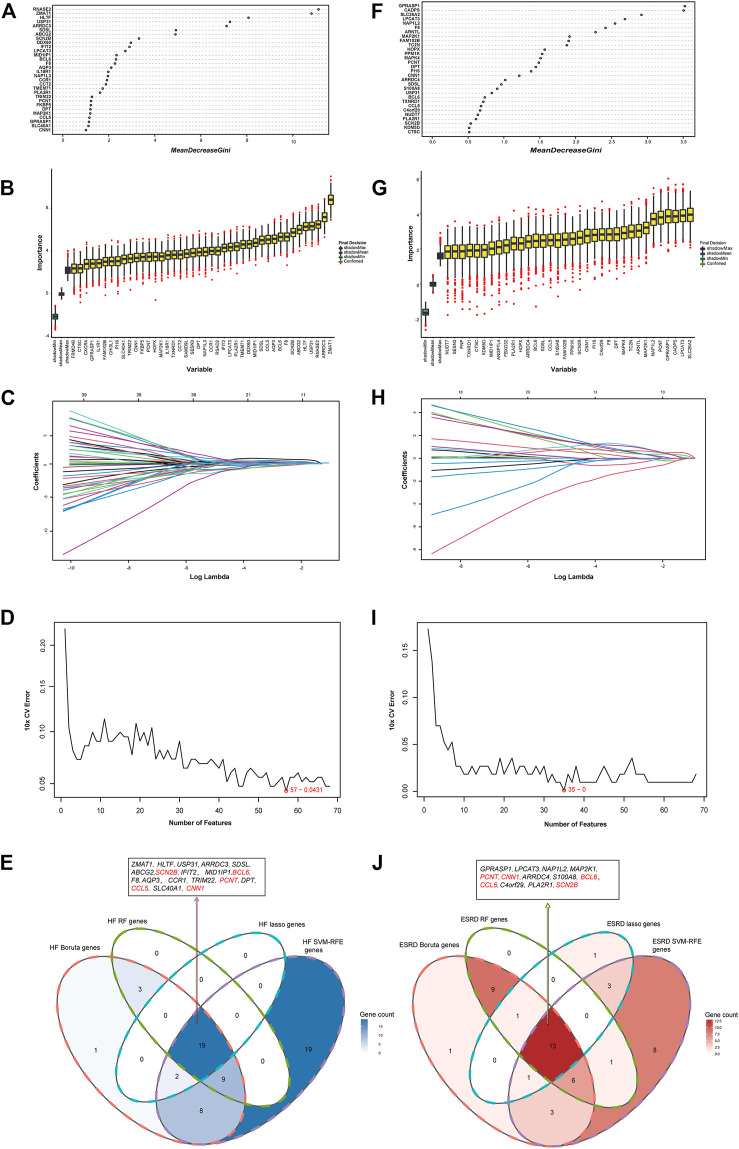
Identification of the hub gene in HF and ESRD datasets. **(A)** RF method identified overlapping DEGs in HF datasets. **(B)** Boruta method identified common DEGs in HF datasets. **(C)** LASSO method identified common DEGs in HF datasets. **(D)** SVM-RFE method identified 68 common DEGs in HF datasets. **(E)** Intersection of common DEGs in HF by the four analyses. **(F)** RF method identified common DEGs in ESRD datasets. **(G)** Boruta method identified common DEGs in ESRD datasets. **(H)** LASSO method identified common DEGs in ESRD datasets. **(I)** SVM-RFE method identified common DEGs in ESRD datasets. **(J)** Intersection of common DEGs in ESRD by the four analyses.

RF, Boruta, LASSO, and SVM-RFE algorithms were used to explore the hub genes shared in HF and ESRD. In the HF dataset, 31 genes were screened by RF algorithm (Figure 5A), 42 genes by Boruta algorithm (Figure 5B), 21 genes by LASSO algorithm (Figure 5C), and 57 genes by SVM-RFE algorithm after tenfold cross-validation (Figure 5D). Eventually, 19 overlapping genes were found in the HF dataset (Figure 5E). In the ESRD dataset, 30 genes were found by RF algorithm (Figure 5F), 34 genes were found by Boruta algorithm (Figure 5G), 19 genes were found by LASSO algorithm (Figure 5H), and 35 genes were found by SVM-RFE algorithm after tenfold cross-validation (Figure 5I). Finally, 13 overlapping genes were found in the ESRD dataset. In the end, the SCN2B, BCL6, CCL5, CNN1, and PCNT genes were found (Figure 5J).

**FIGURE 6:**
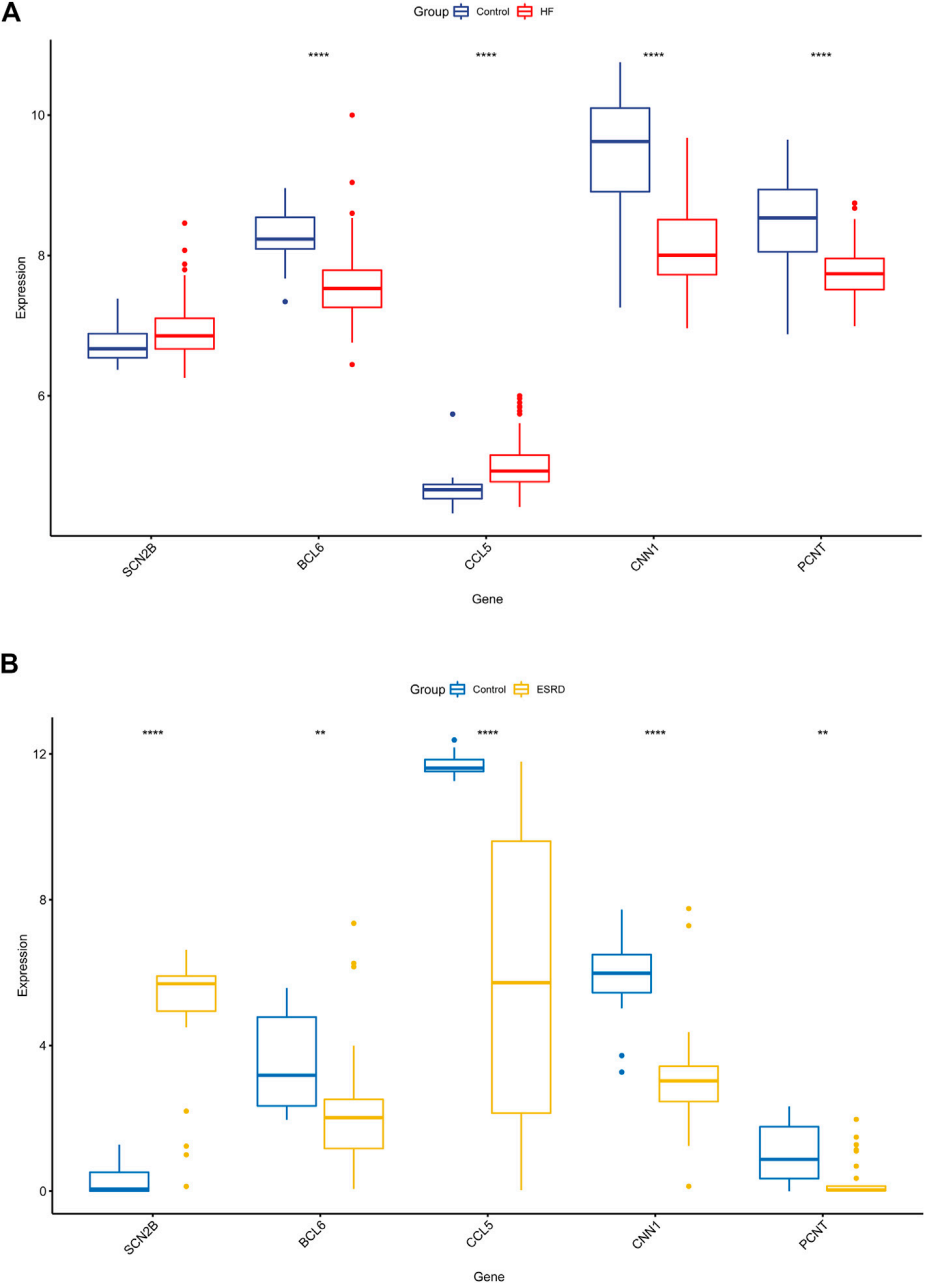
Validation of the hub genes in GSE5406 and GSE97709 datasets. **(A)** Expression levels in GSE5406 datasets. **(B)** Expression levels of the hub gene in GSE97709 datasets. HF, heart failure; ESRD, end-stage renal disease; *p*-values are shown as *, *p* < 0.05; **, *p* < 0.01; ***, *p* < 0.001; ns, not significant.

**FIGURE 7:**
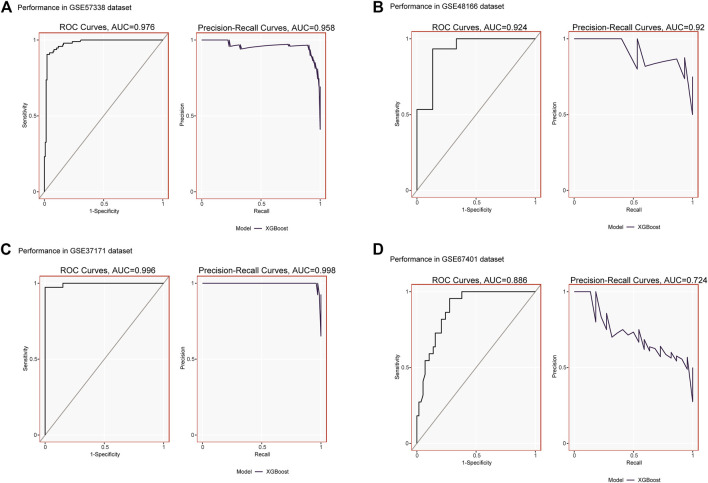
**(A)** XGBoost modeling in the HF training set (GSE57338). **(B)** Validation through the HF validation set (GSE48166). **(C)** XGBoost modeling in the ESRD training set (GSE37171). **(D)** Validation through the ESRD validation set (GSE67401).

We validated the expression of candidate hub genes in the HF dataset (GSE5406) and the ESRD dataset (GSE97709), as shown in Figures 6A, B. However, in the HF dataset, there was no significant difference between the two groups in the expression of SCN2B. Therefore, BCL6, CCL5, CNN1, and PCNT were defined as hub genes in HF and ESRD. Surprisingly, CCL5 expression was increased in HF tissues but decreased in ESRD blood samples. To assess the diagnostic efficacy of the hub genes, we selected one HF dataset (GSE57338) for training and another (GSE48166) for validation. The performance of the training set showed that the values of ROC and PR curves were 0.976 and 0.958 (Figure 7A), respectively, and that of AUC was 0.924 and 0.92 for the validation set (Figure 7B), which illustrated the diagnostic efficacy of the model. To verify its ability to identify ESRD patients, we used the ESRD dataset (GSE37171) for training (ROC = 0.996; PR = 0.998; Figure 7C) and the ESRD dataset (GSE67401) for validation (ROC = 0.886; PR = 0.724; Figure 7D), and the results are also applicable and practical.

### Analysis of immune infiltration in the HF and normal groups

**FIGURE 8:**
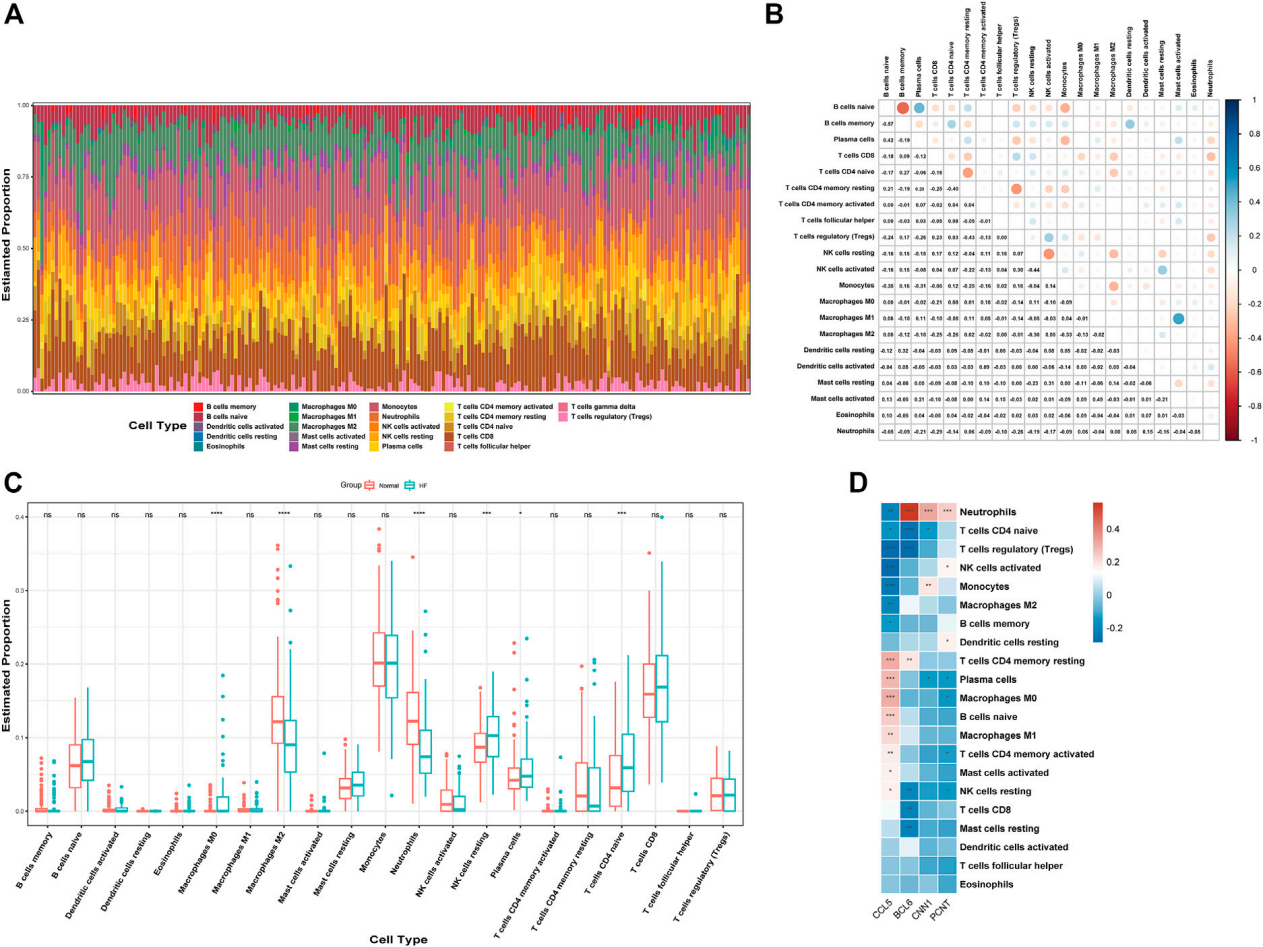
Analysis of immune cell infiltration. **(A)** Relative percentage of 22 immune cell subtypes. **(B)** Correlation heatmap of 21 immune cells. **(C)** Immune cells in HF and normal samples. **(D)** Relationship between hub genes and immune cells related to HF. HF, heart failure; *p*-values are shown as *, *p* < 0.05; **, *p* < 0.01; ***, *p* < 0.001; ns, not significant.

We used CIBERSORT to quantify the enrichment scores of 22 immune cell species and related functions in HF patients and the normal population to explore the differences between myocardial tissue and normal tissue immune cells in HF. The first bar graph clearly shows the proportion of different immune cell subpopulations in each sample (Figure 8A). Regarding the correlation between immune cell subtypes, activated mast cells and M1 macrophages (r = 0.49) have the most significant positive correlation. In contrast, B memory (r = −0.57) cells have the most significant negative correlation (Figure 8B). We find significant differences between HF and normal tissues in the 21 immune cell subpopulations. Therefore, the occurrence of HF is closely related to abnormal immune cell infiltration, and changes in the correlation between immune cells may be related to the occurrence and development of HF (Figure 8C). Specifically, all hub genes showed a remarkable correlation with neutrophil cells (Figure 8D).

### miRNA−mRNA−TF regulatory network construction

**FIGURE 9:**
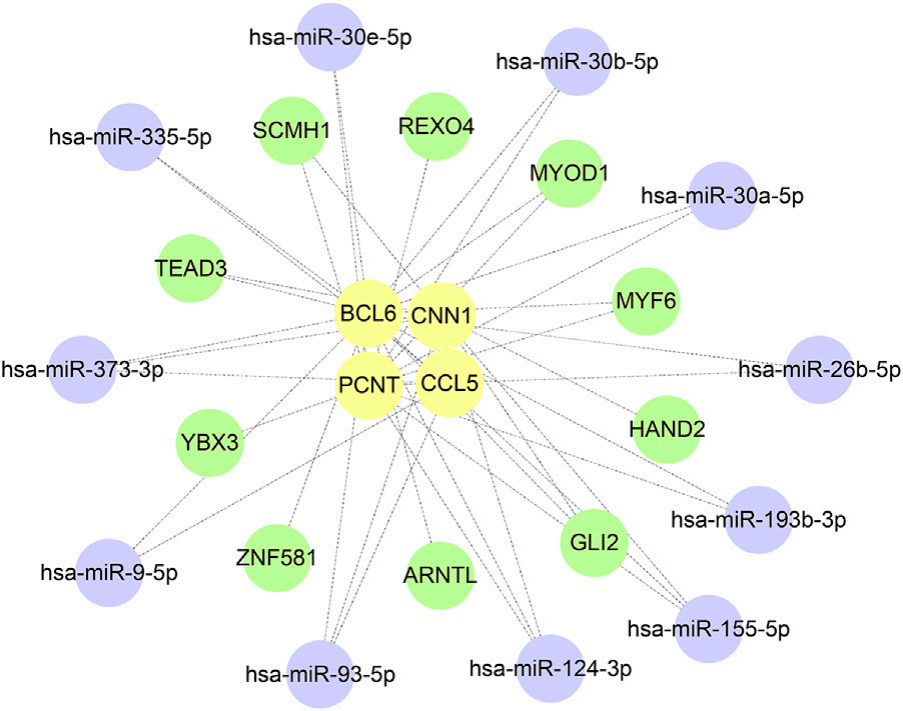
Construction of the hub gene−miRNA network and the target gene−TF network.

A total of 385 miRNA gene pairs were obtained after mapping by three experimentally verified miRNA target prediction databases (Supplementary Tables S1–S3). Finally, 11 overlapping target miRNAs were regarded as key miRNAs. The ChEA3 database was used to enrich TF targets of hub genes to explore their distribution further. The results of CHEA3 prediction showed that SCMH1 ranked the highest among TFs of hub genes (Supplementary Table S4). The top 10 TFs are SCMH1, TEAD3, YBX3, HAND2, MYF6, ARNTL, ZNF581, MYOD1, GLI2, and REXO4. Subsequently, we merged the mRNA−miRNA−TF regulatory network with Cytoscape software (Figure 9).

### Screening for potential pharmacological targets

We used the DSigDB database built on the Enrichr website to search for target drugs for the hub genes. The target drugs for these genes were predicted, and the potential pharmacological target screening was downloaded. The top 10 drug candidates associated with hub genes were selected based on *p*-values and adjusted *p*-values (Table 2).

**TABLE 2:** Candidate drugs of hub genes in the DSigDB.

Index	Name	*p*-value	Adjusted *p*-value	Odds ratio	Combined score
1	Vitamin E CTD 00006994	0.001621	0.049809	36.80624	236.4658
2	Raloxifene CTD 00007367	0.006732	0.049809	28.23392	141.1963
3	Benzene CTD 00005481	0.005436	0.049809	31.6199	164.8915
4	Dasatinib CTD 00004330	0.003648	0.049809	38.992	218.8838
5	Phorbol 12-myristate 13-acetate CTD 00006852	0.003381	0.049809	40.57173	230.8336
6	Digitoxigenin PC3 UP	0.003381	0.049809	40.57173	230.8336
7	AGN-PC-0JHFVD BOSS	5.38E-04	0.04018	104.7989	788.9822
8	Astemizole MCF-7 UP	4.41E-04	0.04018	115.9357	895.7003
9	Vanadic sulfate CTD 00001628	3.64E-04	0.04018	128.0065	1,013.741
10	Etoposide HL-60 UP	3.19E-04	0.04018	136.9034	1,102.189

## Discussion

Patients with HF have benefited from many trials, but patients with ESRD are often excluded from these trials. However, there is little evidence that the benefit of treatment for patients with heart failure combined with advanced kidney disease is altered by the presence or absence of kidney disease. Therefore, more direct evidence is needed to clarify the link between heart failure and end-stage renal disease. In this study, common DEGs and associated pathways between HF and ESRD were explored by bioinformatics analysis. Enrichment analysis revealed that immune and inflammatory responses might play an essential role in HF and ESRD. These findings highlight that immune mechanisms may play a key role in linking HF and ESRD (Rangaswami et al., 2019). Immune cell activation is usually accompanied by the production of inflammatory mediators, such as cytokines, chemokines, and cellular immune receptors, which directly or indirectly affect cardiomyocyte metabolism and promote the development of cardiomyocyte hypertrophy and myocardial fibrosis. GSVA showed that KRAS signaling and TNF-ɑ signaling *via* NF-κB were positively correlated with both HF and ESRD. The KRAS belongs to the Ras gene family, and the role of KRAS in cardiovascular disease was less studied. However, studies have shown a potential role in its association with cardiac cell proliferation and pathological cardiac hypertrophy (Ramos-Kuri et al., 2021). The NF-κB is a transcription factor that has crucial roles in inflammation, cell proliferation, and immunity, and its activation contributes to the pathogenic processes of various inflammatory diseases (Gordon et al., 2011; Song et al., 2019).

Four machine learning algorithms, namely, RF, Boruta, LASSO, and SVM-RFE, were used to screen potential hub genes, and a total of four hub genes were identified to elucidate the crosstalk between HF and ESRD. The XGBoost machine learning model analysis shows that they have a good prediction effect. BCL6 is a transcriptional repressor with anti-apoptotic and pro-oncogenic properties that was initially identified as an oncogene in non-Hodgkin’s B-cell lymphoma. BCL6 is the most strongly characterized inflammatory marker of cardiac aging and predicts the decline in the LV filling rate from early to late stages (Ma et al., 2015). BCL6 is also a regulator of immune cells, and Treg cells lacking BCL6 are uniquely deficient in their ability to suppress Th2 inflammation (Sawant et al., 1950). However, BCL6 may play a role in protecting mature cardiomyocytes from eosinophilic inflammation in myocardial tissue (Yoshida et al., 1999). In cardiac fibroblasts, BCL6 may inhibit cardiac fibroblast activation and function through direct binding to SMAD4 (Ni et al., 2019).

CCL5/RANTES is a T-cell chemotactic agent that is essential for the recruitment of leukocytes to the sites of inflammation, and CCL5 serves as one of the natural ligands for CCR5, which binds to form a family of secreted proteins involved in immune regulation and inflammatory processes (Zeng et al., 2022). CCL5 is thought to drive immune cell migration to the heart tissue of patients with HF, and studies have also revealed an essential role of CCL5 in ventricular remodeling. Stevenson et al. (2019) found significantly increased levels of CCL5 in human hearts with ischemic cardiomyopathy compared to non-failing hearts. Batista et al. (2018) found that in chasmic cardiomyopathy, CCL5 drives the migration of immune cells to myocardial tissue. The protective role of CCL5 in kidney injury has been demonstrated in other models of kidney disease (Krensky and Ahn, 2007). We found that CCL5 expression was increased in the HF dataset but decreased in the ESRD dataset. First, CCL5 in the blood of ESRD patients is influenced by several factors, such as hemodialysis, drug application, and other diseases (Naumnik et al., 2013; Elmoselhi et al., 2016). Second, impaired immune cells in the blood of patients with ESRD may lead to reduced expression of CCL5 (Betjes, 2013). However, this still needs to be confirmed by further studies.

CNN1 encodes a protein that plays a role in regulating and modulating smooth muscle contraction (Takahashi et al., 1986). It inhibits actin-activated myosin ATPase and Ca^2+^-dependent migration of actin. It has therefore been identified as a critical player in the stabilization of actin stress fibers (Liu and Jin, 2016) and its role in cardiomyopathy, placental vascular development, and development of tissue morphology has been revealed. A study of human heart failure gene expression has shown downregulation of CNN1 expression in heart failure, which is consistent with our findings (Tan et al., 2002). In contrast, basic studies found that CNN1 plays an essential role in DCM ventricular remodeling and can inhibit the progression of dilated cardiomyopathy in mice through εPKC signaling (Lu et al., 2014). PCTN is a multifunctional scaffolding protein that binds to various centrosomal proteins (Kim et al., 2019). PCTN regulates many centrosomal functions, such as controlling cell cycle progression, mitotic spindle organization and orientation, and directed cell division. Some studies have shown that it is associated with the development of congenital heart disease (Liang et al., 2020). However, the specific mechanism of the role of CNN1 and PCNT in renal diseases is not well understood and still to be revealed. While our study showed that CNN1 and PCNT are associated with neutrophils and plasma cells, we speculate that it may be related to the differentiation and activation of immune cells in the HF myocardial tissue.

Abnormal immune cells are an important basis for immune dysfunction. Although the quantitative changes and pathway activation/inhibition of these subtypes are not well studied in HF, our study further clarifies the relevant directions. The expression of macrophages, CD4^+^ T cells, NK cells, plasma cells, and neutrophils in myocardial tissue is significantly different from the normal tissue (Li et al., 2021). Consistent with previous findings, M2 macrophages are reduced in heart failure myocardial tissue, which in turn increases cardiac apoptosis and myocardial CD4^+^ T-cell accumulation (Stevens et al., 2022). In contrast, neutrophils exert a deleterious function in an experimental model of heart failure induced by too much pressure (Liu et al., 2021). This finding is consistent with the underlying mechanism of action of neutrophils, which can be involved in the development of multiple cardiovascular diseases through the release of degranulation and recruitment of microvesicles. Therefore, changes in these cell subtypes play an important role in the process of immune response in HF and have significant prognostic and therapeutic value.

It is well known that miRNAs mainly control gene expression, while TFs are involved in target gene transcription. We identified 11 target miRNAs and 10 associated transcription factors to further understand the hub gene associations. Some miRNAs, such as miR-30b-5p (Ren et al., 2021), miR-30a-5p (Qian et al., 2022), and miR-155-5p (Wang et al., 2022), can play a role in the pathological process of HF. The expression of BCL6 can be regulated by the transcription factors TEAD3, MYF6, ARNTL, ZNF581, MYOD1 and GLI2, and TEAD3 (Han et al., 2020). GLI2 (Voronova et al., 2012) plays an essential role in embryonic myocardial development and cardiomyogenesis. Meanwhile, to predict potentially effective therapeutic agents, we applied the DSigDB to identify 10 possible therapeutic agents. Among them, vitamin E can reduce the activation of inflammatory factor NF-қB, which leads to cytokine/chemokine and mast cell activation, and has been confirmed in related studies (Tettamanti et al., 2018). However, the molecular pathways and potential therapeutic compounds screened by our bioinformatics approach still need further validation by cellular experiments and clinical samples.

## Conclusion

To conclude, we identified BCL6, CCL5, CNN1, and PCNT as hub genes between HF and ESRD. This finding contributes to understanding the close interrelationship in the development of ESRD and HF. This study also provides some theoretical basis for the possible search of new drug targets and developing new therapeutic approaches.

## Data Availability

Publicly available datasets were analyzed in this study. These data can be found at: GEO database (https://www.ncbi.nlm.nih.gov/geo/), accession numbers GSE57338, GSE5406, GSE135055, GSE37171, and GSE97709.

## Abbreviations

AUC, area under the curve; Boruta, Boruta algorithm; CHEA3, ChIP-X Enrichment Analysis 3; DEG, differentially expressed gene; DSigDB, Drug Signatures Database; ESRD, end-stage renal disease; GEO, Gene Expression Omnibus; GSVA, gene set variation analysis; GO, Gene Ontology; HF, heart failure; KEGG, Kyoto Encyclopedia of Genes and Genomes; LASSO, logical regression of the selection operator; PPI, protein−protein interaction; PR, precision–recall; RF, random forest; ROC, receiver operating characteristic; SVM-RFE, support vector machine-recursive feature elimination; XGBoost, eXtreme Gradient Boosting.
